# Relational Morphology: A Cousin of Construction Grammar

**DOI:** 10.3389/fpsyg.2020.02241

**Published:** 2020-09-23

**Authors:** Ray Jackendoff, Jenny Audring

**Affiliations:** ^1^Center for Cognitive Studies, Tufts University, Medford, MA, United States; ^2^Gibson/Fedorenko Lab, Department of Brain and Cognitive Sciences, Massachusetts Institute of Technology, Cambridge, MA, United States; ^3^Leiden University Centre for Linguistics, Leiden University, Leiden, Netherlands

**Keywords:** relational morphology, construction grammar, parallel architecture, lexicon, language processing, schema, interface, words and rules

## Abstract

Relational morphology (RM) is a novel approach to word structure that bears a close relation to construction grammar (CxG). Based on the parallel architecture framework, its basic question is: what linguistic entities are stored in long-term memory, and in what form? Like CxG, RM situates the “rules of grammar” in an extended lexicon, right along with words, multiword expressions such as idioms and collocations, and meaningful syntactic constructions. However, its notion of *schema* enriches CxG’s notion of *construction* in a number of respects, including (a) the possibility of purely formal schemas that lack meaning, (b) a more precise way of specifying relations among lexical items than standard inheritance, (c) the possibility of “horizontal” relations between individual words and between schemas, (d) a clearer characterization of the distinction between productive and nonproductive phenomena, and (e) more explicit integration with theories of language processing and of other domains of cognition.

## Introduction

Over the past decade, we have been developing an approach to linguistic structure called *relational morphology* (RM; Jackendoff and Audring, 2020). Its goal is a graceful integration of morphology with the rest of language and with the rest of the mind. RM is conceived of as a component and an enrichment of the *parallel architecture* (PA; Jackendoff, 1997, 2002, 2011); other major components are *conceptual semantics* (Jackendoff, 1983, 1990, 2007a) and *simpler syntax* (Culicover and Jackendoff, 2005). The present article is drawn primarily from Jackendoff and Audring (2020).

Relational morphology takes very seriously the term “knowledge of language,” focusing on the question of what a speaker stores in long-term memory and, crucially, in what form. The outcome is a conception of language quite different from mainstream generative grammar, including the minimalist program (Chomsky, 1965, 1981, 1995; Berwick and Chomsky, 2016; for comparison, see works cited above). RM might be considered a close cousin of construction grammar (CxG; Goldberg, 1995, 2006, 2019; Croft, 2001; Hoffmann and Trousdale, 2013) and construction morphology (CxM; Booij, 2010, 2018). In some respects, it is related to HPSG (Pollard and Sag, 1994) as well. Anticipating the discussion to follow, the areas of consilience (and of contrast with generative approaches) include the following:

• “Rules of grammar” are stated as declarative *schemas* (a.k.a. *constructions*) rather than as procedural rules.
• “Rules of grammar” are in the same basic format as words: structured relations of form and meaning. Hence there is no distinction between the “lexicon” and the “grammar”; both words and rules are treated as items in an “extended lexicon” or “constructicon.”
• The basic combinatorial operation is Unification.
• Relations among lexical items are stated in terms of inheritance.
• Language acquisition is item-based.

On the other hand, there are some important differences between PA/RM and CxG. First, most varieties of CxG define a construction as an association between a form (syntax and phonology) and a function (semantics), i.e., a Saussurian sign. The PA, like CxG and unlike traditional generative grammar (including the minimalist program), argues that the grammar must include constructions that specifically link syntactic form to meaning, such as the *way*-construction (e.g., *Harry hiccupped his miserable way down the hall*). But it also admits the possibility of schemas/constructions that do not involve semantics, for instance phrase structure rules, phonotactics, meaningless morphological elements such as linking elements in compounds, and grammatical “glue” such as *do*-support and the *of* in *picture of Bill*. RM further extends the use of schemas to phenomena where meaning plays no role, such as the relation of phonology to orthography and the relation of poetic texts to a metrical grid (Jackendoff and Audring, 2020, ch. 8). Hence the PA views constructions that relate form and function as only a subset of a speaker’s full knowledge of language.

A second difference between PA/RM and standard CxG concerns the repertoire of relations among lexical items. As mentioned above, the principal type of lexical relation in CxG (and HPSG) is *inheritance*, a relation between a word or construction and another, more abstract construction, such that the latter partially motivates the structure of the former. PA/RM admits such relations, but in addition it countenances direct “horizontal” or *sister* relations among words or among schemas, for which in many cases it is unattractive to posit an abstract “mother” that captures what they have in common. We illustrate below.

A third difference between the frameworks is in the formalism. We adopt the PA/RM formalism over the attribute-value matrices of HPSG and formal CxG, partly because it is easier to read, but also, more importantly, because it stresses the distinction between phonology, syntax, morphosyntax, and semantics. It also provides a way to exactly pinpoint what related items have in common, as well as a way to distinguish productive from nonproductive patterns. Again, these points will emerge from the discussion below.

A final, more philosophical point of divergence is that PA allows for the possibility of domain-specific principles of language, while CxG tends to view language entirely as a byproduct of more domain-general cognitive processes. PA is of course committed to minimizing the domain-specific aspects of language, but it does not assume them to be zero.

We believe that these points of difference can easily be grafted onto more standard versions of CxG, as what RJ’s colleague Dan Dennett calls “friendly amendments.” We are not going to specifically argue for these points. Rather, we wish to offer a general feel for the PA/RM framework, while pointing out the similarities to CxG – and the differences – as we go along.

Section “Parallel Architecture Basics” lays out the basic constructs of PA/RM, sections “Words and Rules in the Lexicon” and “‘Bound Roots,’ Sisters and Sister Schemas” lay out the RM approach to a number of morphological phenomena, in particular the use of schemas both to generate novel forms and to establish explicit relations among items stored in the lexicon. Section “Sister Schemas in Phrasal Syntax” sketches extensions of the latter function to syntactic phenomena. Section “Lexical Access in the Extended Lexicon” shows how the constructs of RM can be incorporated directly into a theory of language processing. Finally, section “Beyond Language” suggests that these constructs are useful in thinking about memory in other cognitive domains such as music and the conceptualization of physical objects.

## Parallel Architecture Basics

**FIGURE 1:**
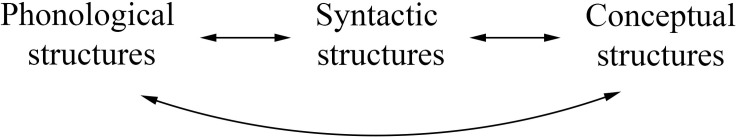
The parallel architecture.

The most basic premise of the PA is that linguistic structure is not determined entirely by syntax, as it always has been in standard generative grammar. Nor is it determined entirely by meaning, as some practitioners of cognitive grammar advocate. Rather, linguistic structure is determined by independent systems for phonology, syntax, and semantics, plus the linkages (or *interfaces*) between them, as in Figure 1. The double-headed arrows in Figure 1 are meant to represent *correspondences* between components rather than *derivations* from one component to another.

A well-formed sentence has well-formed structures in each domain, plus well-formed links among the structures.

**FIGURE 2:**
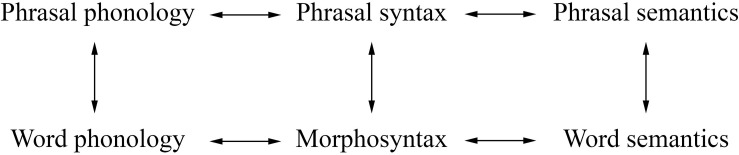
The parallel architecture, incorporating relational morphology.

Following the lead of CxM, RM treats morphology as the grammar of words (Booij, 2012). Like phrasal grammar, morphology involves phonology, syntax, and semantics, but *inside* of words. Thus the picture in Figure 1 can be elaborated along the lines of Figure 2.

In the RM architecture, then, morphology encompasses the structure of morphosyntax, plus its interfaces to phrasal syntax and to the phonology and semantics of words. (Antecedents for this view include Bach, 1983; Ackema and Neeleman, 2004; Van der Hulst, 2006.)

On this view, there is a degree of continuity between morphosyntax and phrasal syntax. For instance, both involve X^0^ syntactic categories and headed hierarchical structure, and both deal with inflectional categories such as number and case, though in different ways. On the other hand, there are differences as well: only phrasal syntax has XPs and long-distance dependencies; while (arguably) only morphosyntax has affixes and such phenomena as noun incorporation and templatic inflection. In other words, morphosyntax does not reduce to a form of phrasal syntax, as it does in, say, distributed morphology (Embick and Noyer, 2007; Siddiqi, 2019). But it is also not entirely distinct from syntax, as it is in, say, paradigm function morphology (Stump, 2019).

An important corollary to this conception of grammatical architecture is that it extends naturally to the relation between language and other cognitive domains. For instance, in order to talk about what we see, there has to be an informational conduit between high-level visual representations and linguistic semantics. It cannot be modeled in terms of a derivation from syntax to vision or vice versa, but it can be readily envisioned as a set of correspondences linking visual representations and linguistic structures.

Where is the lexicon in this architecture? For the simplest sort of example, a word such as *cat* consists of a piece of semantic structure (the meaning of the word), a piece of phonological structure (/kæt/), and the syntactic category Noun. The bundling of these components into a lexical unit is conventionally notated by enclosing them in large square brackets. We instead notate their relation by co-subscripting, as in (1). The subscripts can be thought of as marking the ends of association lines between the components; in other words, they denote what we will call *interface links*. (Jackendoff, 1997, 2002 calls them *correspondence rules*.)


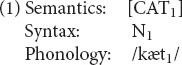


A word, then, consists of a collection of representations, linked across the three levels. Hence the lexicon, where stored words live, cuts across multiple components of the architecture in Figure 2 – including the links *between* components.

This conception of words has an immediate interpretation in terms of language processing. In comprehension, upon hearing /kæt/, the existence of (1) allows the hearer to posit a noun and the meaning CAT in the linked syntactic and semantic structures under construction. In production, (1) allows the speaker to express the intended message CAT as a noun pronounced /kæt/. There is nothing very unusual here, except that the distinctions among levels and the links among them are foregrounded. We return to processing below.

## Words and Rules in the Lexicon

An important respect in which constraint-based theories such as CxG, CxM, HPSG, and the PA differ from traditional generative linguistics is the status of rules of grammar. For instance, consider the regular plural in English. In a traditional generative grammar, the formation of plurals is governed by a *rule* roughly of the form “To form the plural of a noun, add *-s*.” The counterpart in the PA is a *schema* of the form (2); CxG and CxM would think of it as one way of formalizing the “English plural noun construction.”


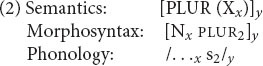


Like the word in (1), schema (2) consists of a piece of semantics, a piece of morphosyntax, and a piece of phonology; the three are linked by subscripts. (2) differs from (1) in that parts of its structure are variables: it says that a multiplicity (PLUR) of any sort of entity (X) can be expressed by a noun (N) plus a plural affix (PLUR); in phonology, the combination is pronounced however that noun is pronounced, followed by the phoneme /s/. Given this schema, the plural form *cats* can be produced by instantiating the variables in (2) with the corresponding pieces of (1), resulting in the structure (3). (2) can be similarly instantiated with newly encountered nouns, to spontaneously produce novel expressions such as *wugs* and *coelacanths*.


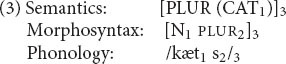


The parallel architecture, like CxG and CxM, proposes that all rules of grammar are to be stated in schema (i.e., constructional) form: they are in essentially the same format as words, except that some of their structure is made up of variables. Blurring the distinction further, a verb’s subcategorization feature amounts to a syntactic variable that must be instantiated; and a selectional restriction similarly amounts to a semantic variable.

This approach to rules of grammar extends even to syntactic phrase structure rules, such as that for the English transitive verb phrase, approximated in (4). This is a piece of linguistic structure that involves only one level of structure and that consists *entirely* of variables. One can think of it as a “treelet” in the sense of Fodor (1998) and Tree-Adjoining Grammar (Joshi, 1987).


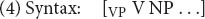


Lacking intrisic meaning, a schema like (4) is not generally countenanced in CxG, but CxG can easily assimilate it by relaxing the stricture that every construction is a form-function pair. This seems correct. The fact that English canonically has SVO order while Turkish has SOV order does not reflect any difference in meaning between the two languages. It is a purely syntactic fact.

In essence, then, there need be no further distinction between lexical structure and rules of grammar: they belong together in a single system that might be called the “extended lexicon”; CxG sometimes uses the term “constructicon” in the same sense (Hoffmann and Trousdale, 2013). Schemas fulfill the traditional function of rules – creating an unlimited number of novel structures – through the operation of *unification* (Shieber, 1986). Unification instantiates a schema’s variables with further phonological, syntactic, and/or semantic material, as seen in the structure of *cats* above. Hence, the composition of a word or sentence involves clipping together stored pieces in such a way that every element of the composed structure is accounted for in terms of one stored piece or another. A schema that permits the productive instantiation of its variable(s) serves what we call a *generative function*.

However, unlike traditional rules, schemas have a second function, which we will call the *relational function*. This function is often implicitly invoked for CxG’s constructions, though it is not usually recognized as distinct from the generative function. In order to explain the relational function, we first have to supplement interface links with a second sort of links: *relational links*.

Toward that end, consider a pair of words like *hate* and *hatred*. The string -*red* looks like a deverbal suffix tacked onto *hate.* However, *hatred* is the only word with this suffix. Other such cases of words with unique affixes include *bombard*, *comparison*, *knowledge*, and *laughter*. (They are admittedly rare.) It would be peculiar to posit a traditional word formation rule along the lines of “to form a noun based on *hate*, add -*red*.” A rule that applies only to a single item is no rule at all. However, the relation between *hate* and *hatred* can be captured in the RM notation, as shown in (5) (semantics approximate).


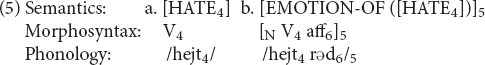


Subscript 4 links the three levels of *hate*, and similarly, subscript 5 links the three levels of *hatred*. But subscript 4 also links *hate* to the base of *hatred*, marking the two as the same. This connection is what we call a *relational link*. This link is not used to derive *hatred*; rather, it simply marks what the two lexical items share. The link thereby “supports” or “motivates” *hatred*: it makes it less arbitrary than a word such as *ibex* that lacks internal structure. *Hatred* is easier to learn, then, because it has a previously known part; and it is easier to process, because of the extra activation that comes from *hate*.

We note that CxG typically does not concern itself with relations between words like *hate* and *hatred*. The focus tends to be inheritance relations between either words or constructions and more abstract constructions. Nevertheless, it would not be difficult to add word-to-word relations to the CxG lexicon. We elaborate this point below.

We now return to the functions of schemas. They too can take part in relational links. Consider the idiomatic expressions in (6), which all contain the plural -*s* suffix.

(6) raining cats and dogs, odds and ends, best regards, make   amends, …

The meanings of these expressions cannot be built up from the meanings of their parts, so the expressions must be learned and stored. But that does not entail that they are stored as holistic unstructured units. In particular, the plural nouns are still standard plural nouns, even though they are not spontaneously generated. RM captures this generalization by establishing relational links between the plural schema (2) and these idiomatic stored plurals. In this case, the connection is not between shared subscripts, but rather between variable subscripts in the schema and constant subscripts in its instances. Again, the intuition is that the relational link to the schema makes these idioms easier to learn and process.

There is an important consequence: a schema can be used not only in a generative function, to create novel structures, but also to motivate items that are stored – its *relational function*. The novel plural in *wugs* and the stored plurals in *raining cats and dogs* invoke the very same plural schema (2), just used differently: generatively in the former case and relationally in the latter. A similar situation arises with the transitive VP schema (4): it is used generatively in novel VPs such as *throw the pail*, but also relationally, in VP idioms such as *chew the fat*. This twofold use of schemas contrasts with the rules of traditional rule-based approaches, which play only a generative role. Idiomatic uses of productive patterns, if addressed at all, are often dismissed as unsystematic exceptions (see Jackendoff and Audring, 2020, section 2.1 for examples).

We next observe that not all schemas have these two uses. Many of them can perform only the relational function. An example is the family of deadjectival verbs such as *darken*, *widen*, *harden*, *tighten*, and *sharpen*. There is clearly a pattern: an adjective serves as base; it is followed by the affix -*en*; and the composite is a verb that means “(cause to) become A.” This family is supported by schema (7).


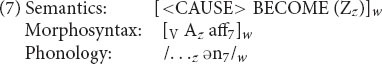


Unlike the plural schema, (7) is at best uncomfortable with the generative function. English speakers do not produce or accept novel applications of (7) such as ^∗^*louden* (“make/get louder”), ^∗^*crispen*, or ^∗^*colden*, as the generative function would predict. Apparently, (7) can be used only to motivate items that are stored, i.e., it has only the relational role. Such patterns are the norm in English derivational morphology, rather than the exception.

Nonproductive patterns occur in syntax as well, though not as pervasively as in morphology. (8) Illustrates two of them; more such “syntactic nuts” appear in Culicover (1999).


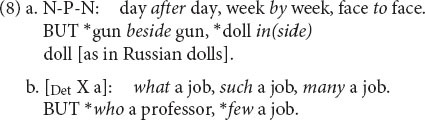


In the N-P-N construction, the choice of possible prepositions is fixed and must be learned; in the determiner construction, the set of possible “predeterminers” is similarly idiosyncratic. Yet there is a clear pattern, captured by a schema that can only be used relationally.

To sum up, the extended lexicon is a single system that stores not only words, but also grammatical schemas. Like CxG and HPSG, it also stores idioms and common collocations such as *red as a beet* and “prefabs” such as *I think so* (Wray, 2002; Jackendoff, 1997). Under this conception, the traditional role of rules of grammar is taken over by schemas, employed in their generative function. In turn, schemas are stated in the same terms as words, namely as pieces of linguistic structure – semantics, (morpho)syntax, and phonology – connected by interface links where appropriate. They differ from words in that they have variables that must be instantiated in constructing an utterance.

Relations among stored words are expressed by relational links, which mark parts of related items as the same. Relational links also connect schemas to their stored instances. In this role, they do the work traditionally ascribed to nonproductive lexical redundancy rules. Furthermore, many schemas have *only* the relational function.

Reframing these conclusions, it becomes evident that *all* schemas can function relationally, while only *some* schemas can function generatively. One can think of the latter, then, as schemas that have so to speak “gone viral.”

The formal distinction between productive and nonproductive schemas is marked on the schemas’ variables: a “closed” (i.e., nonproductive) variable requires its instances to be listed in the lexicon, while an “open” (productive) variable can be freely applied to produce novel instances. There may be a cline between fully closed and fully open variables, with intermediate cases that allow new instances under special conditions (e.g., *Trumpification*). (It is unclear to us exactly what factors lead a language learner to determine whether a schema is productive or not; for discussion, see Jackendoff and Audring, 2020, pp. 45–50, 228–231.)

This treatment of the distinction between productive and nonproductive schemas has major consequences for linguistic theory. First, it eliminates the distinction between “grammatical rules” and “lexical rules” as separate components of grammar; the difference is reduced to a featural distinction within schemas. A larger moral is that the focus of linguistic theory ought to expand beyond the subset of generative patterns to encompass all patterns, productive or not. Again, it is not hard to envision an enrichment of standard CxG that incorporates these innovations.

## “Bound Roots,” Sisters and Sister Schemas

With this sketch of PA/RM in hand, we next illustrate some of the descriptive power of the RM formalism. This will put us in a position to think about some broader questions in the next sections, as well as some challenges for standard CxG.

A first case is words that have a well-established suffix attached to a base that is not a word on its own. These are sometimes called “bound roots.” They are often noticed in the literature, only to be quickly set aside as a minor glitch in the system. But in fact English has hundreds of them, for instance those in (9), so the theory disregards them at its peril.


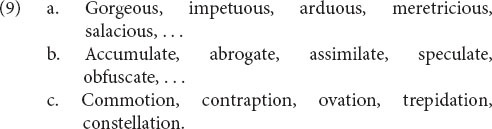


Here is the structure of *gorgeous*.


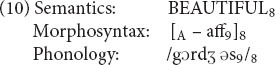


The affix -*ous* in phonology is marked in morphosyntax as an affix (coindex 9). But morphosyntax has nothing that can link to the *gorge* part, since *gorge* (in the relevant sense) is not a word on its own and hence has no syntactic category. We notate this absence of morphosyntax with a dash. Furthermore, the meaning of *gorgeous* cannot be divided into the meaning of the base plus the meaning of the affix, so semantics doesn’t have any internal links either. Hence the internal linkages are confined just to the structure of the affix. We think this is exactly what one would want to say about the structure of this word. To use Anderson’s (1992) term, *gorgeous* is partly a-morphous: -*ous* is a morpheme but *gorge*- is not.

Next we need to say that there are a lot of these -*ous* words with bound roots. This can be expressed with a schema along the lines of (11).


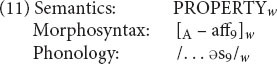


The semantics of (11) says that an -*ous* word denotes a property, which is essentially the basic meaning of any adjective. Morphosyntactically and phonologically, the whole word is an adjective that ends in an affix, pronounced /əs/ (coindex 9). And that’s all: (11) says nothing about the form, the syntactic category, or the meaning of the base.

For the next case, consider the relation between *assassin* and *assassinate*. From a morphological perspective, *assassin* is contained in *assassinate*, just as *hard* is contained in *harden*. But from a semantic perspective, an *assassin* is someone who assassinates people, so the meaning of *assassinate* is contained in that of *assassin*. This presents a paradox to traditional word-formation rules, since the “direction of derivation” is mixed: neither can serve as the base for the other. It also presents a difficulty for the traditional view of inheritance as an asymmetrical relation, “X inherits structure from Y.” In the case of *assassin* and *assassinate*, each word has to inherit part of its structure from the other.

The RM notation overcomes this problem by formulating their relation as (12). (The semantics is very approximate.)


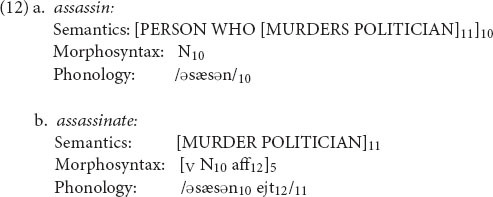


Here, both words contain the phonological base *assassin*, coindexed 10; this is the whole of *assassin* and part of *assassinate*. Similarly, both words contain the semantics “murder a politician,” coindexed 11; this piece of semantics forms part of *assassin* and the whole of *assassinate*. Thus the notation captures exactly what one would want to say about this pair. We’ll use the term *sisters* for such pairs of words, in which neither can be derived from the other, and there is no overarching “mother” that they can both inherit from. Other examples of this sort are *critic ∼ criticism* and *linguist ∼ linguistics*.

Another sort of sister relation appears in (13): *ambition* and *ambitious* share a nonlexical base but have different affixes. Other such pairs include *contagion ∼ contagious* and *cognition ∼ cognitive*.


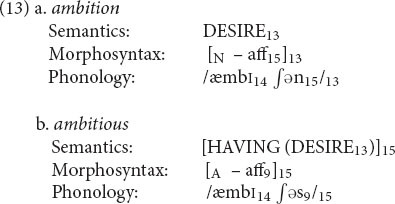


As with *gorgeous*, the morphosyntax of the base has no syntactic category and is not linked to anything. However, the two words share the phonology of the base (coindex 14); and the meaning of *ambition* is contained in the meaning of *ambitious* (coindex 13). Finally, each of the words has its own affix (coindices 15 and 9, respectively).

A traditional treatment of these words in terms of word formation rules would have to capture the relation between them by positing an abstract form *ambi(t)* from which the two words would be derived. This form would somehow have to stipulate that it can be pronounced only if attached to either -*tion* or -*ous*, a highly artificial solution. Alternatively, it might be proposed that *ambitious* is derived from *ambition* by deleting -*tion* to form *ambit-* and then appending –*ous* – an equally ugly solution. An account in terms of inheritance would similarly require an abstract construction that contributed */æmbi(t)/* and DESIRE but left the affix open, yet again not an optimal solution. In contrast, the sister relation in the RM formalism again says exactly what needs to be said.

(12) and (13) illustrate sister relations between *words*. The story gets more interesting when we consider what Booij (2010) and Booij and Masini (2015) call *second-order schemas* and what we call *sister schemas*: pairs of systematically related patterns, as in (14). Each individual pair is connected by a shared base. Some of the shared bases are independent words (14a), and some are not (14b). (To be sure, some of the relations in (14b) are historically derived from Latin or Italian roots. However, we are modeling the synchronic knowledge of an ordinary speaker who has no awareness of their etymology.)


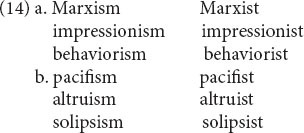


As with *ambition/ambitious*, the relation is bidirectional. The left-hand member of each pattern contains the suffix *-ism* and denotes an ideology or world view, while the right-hand member contains the suffix *-ist* and denotes an adherent of that very ideology or world view. The *-ism* member of the pair can be considered “primary” in the sense that its meaning is contained in that of the corresponding -*ist* word. But *deriving* the -*ist* word from the -*ism* word faces the same difficulties as deriving *ambitious* from *ambition* or vice versa.

This interweaving of patterns is formulated as the sister schemas in (15).


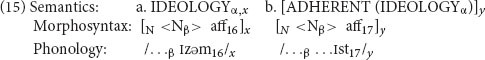


(15a) is the schema for the left-hand words in (14); variable subscript *x* links the semantics, morphosyntax, and phonology, as usual. The morphosyntax and phonology of the suffix -*ism* are linked by coindex 16. Similarly, (15b) is the schema for the right-hand words, with variable subscript *y* tying the three levels together; and the suffix *-ist* is tied together by coindex 17. The optional N in morphosyntax is present for the cases like (14a) with a lexical base; if it is absent, we get the cases with a nonlexical base such as (14b).

So far, then, we have two independent schemas, (15a) for the -*ism* words and (15b) for the -*ist* words. However, there is more to say, namely that the two schemas are related to each other as sisters. This relation is notated by the Greek letter coindices α and β, which denote variables that are shared between the two schemas. They say that if there is a word that denotes an ideology and ends in -*ism*, there is likely to be a word that has the same phonological base (coindex β), denotes an adherent of that same ideology (coindex α), and ends in *-ist*. In other words, Greek letter coindices denote a third sort of relational link, in addition to links between words and links between a schema and its instances. (Note that -*ist* words that do not denote ideologies do not participate in this alternation. For instance, the fact that there is a word *trombonist*, denoting a person who plays the trombone, does not motivate a possible word ^∗^*trombonism*.).

Sister schemas prove to be ubiquitous in such morphological phenomena as paradigmatic relations, inflectional categories, stem allomorphy, reduplication, and systematic truncation (Jackendoff and Audring, 2020, chapters 4–6). Moreover, it is significant that the treatment of sister schemas is a simple formal extension of sister *words*. This constitutes another reason for eliminating the distinction between the lexicon and the grammar, treating both as denizens of the extended lexicon, with similar formal properties. And again, the notion of sister schemas is available neither to traditional word-formation rules nor to standard notions of inheritance.

## Sister Schemas in Phrasal Syntax

Thinking more broadly, we might ask whether phrasal syntax also makes use of sister schemas. The appropriate configuration would be two constructions (a) which share significant structure, but (b) neither of which can be derived from the other, and (c) for which it is implausible to posit a common “mother” construction from which both can be derived (or, in CxG terms, from which both can inherit). Such configurations have been introduced tentatively in CxG (Cappelle, 2006; Van de Velde, 2014; Zehentner and Traugott, 2019). In fact, they were recognized by Harris (1957), whose notion of transformation amounted to a systematic correspondence between two patterns – quite different from the transformations in his student Chomsky’s (1957) *Syntactic Structures*.

One example of this sort of relationship is the English particle alternation: *look up the answer* vs. *look the answer up*. Cappelle (2006), in a CxG framework, treats these as “allostructions” of a more abstract common mother. In the RM formalism, the common mother is unnecessary. Rather, the two alternatives are treated as sister schemas, as in (16). (See Audring, 2019 for a fuller discussion of when sisters suffice and when a “mother” is needed.)


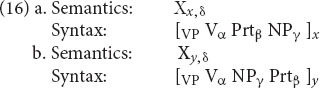


These schemas share their meaning (linked variable δ), their verb (linked variable α), their particle (β) and their object (γ); they differ only in the linear order of the latter two. Through the linking of the variables, the schemas in effect say that if a verb and particle appear in one of these patterns, they can be predicted to appear in the other.

Another candidate for sister schemas, also involving particle verbs, is their relation to their nominalizations, as in (17) (see Booij and Masini, 2015 for parallels in Dutch).


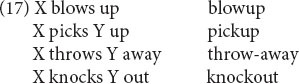


The nominals have the usual semantics for nominals: either the process involved in the event denoted by the verb (e.g., *blowup* denotes the process or action of blowing up) or the Patient of the event denoted by the verb (e.g., *throw-away* denotes something that is thrown away). However, as shown by Chomsky (1970), it is impossible in general to derive the nominals from the verbs; and it is not clear how one would formulate a more abstract form or construction from which both forms could inherit. (18) shows how the syntactic part of the relation can be formulated in terms of sister schemas.


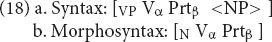


Here the verb and particle are shared (coindices α and β). But in (18a) they are combined into a VP with a possible direct object, while in (18b) they are combined into a noun. In other words, the sister relationship here is between a phrasal schema and a morphological schema.

Jackendoff and Audring (2020) analyze numerous syntactic, morphological, and phonological phenomena in terms of sister schemas. Accordingly, we find it plausible that all alternations that have been formulated in terms of derivations can instead be treated in terms of sister schemas – in other words, Harris was right! Exploring this possibility is a major undertaking for future research.

## Lexical Access in the Extended Lexicon

One desideratum for the PA and RM is that they should make contact with issues in language processing. To that end, we sketch how the PA/RM theory of linguistic representations can be embedded in a theory of language processing. The overall burden of the argument is that constructs that are familiar from standard accounts of processing can be interpreted readily in the terms of the representational theory. Hence PA/RM allows for a graceful connection between competence and performance. (For more detail, see Jackendoff, 2002, 2007b; Jackendoff and Audring, 2020; Huettig, Audring, and Jackendoff, Prediction as pre-activation: a linguistically and psychologically plausible theory of language processing, in preparation.)

To recapitulate, a fundamental point of PA/RM is that schemas are stored in the extended lexicon, right alongside of words. Both consist of pieces of linguistic structure – stored declarative knowledge – and both involve interface links that connect their levels, as well as relational links to other lexical items. The consequence for processing is that all principles of lexical activation and lexical access apply to schemas in the same way as they apply to words. This is not possible in traditional accounts, in which the lexicon and the grammar are quite distinct. For instance, while the character of lexical access is taken to be a central concern, the literature does not typically recognize the parallel issue of “rule access,” i.e., choosing what rule to apply in a derivation. Rather, standard accounts are stated in terms of choosing among *structures* – the *outputs* of rule application, such as high vs. low PP attachment in *the woman saw the man with a telescope*. This is quite a different process from accessing words. By contrast, in the PA/RM framework, constructing or parsing a sentence involves activating and selecting pieces of structure such as the transitive verb phrase schema – through the very same process that activates and selects words. Thus choosing among structures is altogether natural.

A second basic point is that the brain does not explicitly keep track of the frequency of lexical items. Rather, in concurrence with much of the psycholinguistic and neuroscience literature (e.g., Collins and Loftus, 1975; Bybee, 1995; Baayen et al., 1997; Pinker, 1999), we take frequency in a corpus as a proxy for “resting activation.” Any use of a word augments its resting activation, such that it responds more quickly and/or more robustly to further activation (e.g., McClelland and Elman, 1986; Norris et al., 2000), such that it can (somewhat stochastically) outcompete other items for “what is being heard” (e.g., Marslen-Wilson, 1987; O’Donnell, 2015).

Putting these two points together, it follows that more frequent *schemas* (e.g., more frequent syntactic constructions) likewise have a higher resting activation than less frequent ones, making their response more robust in both comprehension and production. This appears to be in line with evidence in the psycholinguistic literature (e.g., MacDonald et al., 1994).

The general course of processing takes place along lines suggested earlier for the comprehension and production of *cat* in example (1). In language comprehension, phonological input leads to activation of identical (or sufficiently similar) pieces of phonological structure in the lexicon (Marslen-Wilson and Tyler, 1980; McClelland and Elman, 1986). These in turn pass activation – through interface links – to corresponding syntactic and semantic structures in the lexicon. The processor attempts to integrate these structures with the syntactic and semantic structures that have been built so far on the basis of previous input.

Language production is the mirror image: the desired message, encoded in semantic/conceptual structure, activates identical (or sufficiently similar) pieces of semantic structure in the lexicon. These in turn pass on activation to corresponding syntactic and phonological structures in the lexicon (Levelt et al., 1999), through interface links. The processor then attempts to integrate these structures with the syntactic and phonological structures built in response to earlier parts of the intended message.

Activation does not just spread “vertically,” via interface links, to other levels of representation. It also fans out “horizontally,” in what the literature generally calls spreading activation (Collins and Loftus, 1975): activation of one item activates similar or related items. However, we can be more precise: activity spreads specifically through relational links. Hence the intensity of activity that spreads from one item to another is determined not only by the level of activation of the “donor” item, but also by the degree to which the items in question are linked relationally. Therefore, we predict that more activation will be spread between items whose relation is relatively transparent, such as *joy/joyous*, compared to a less closely related pair such as *malice/malicious*, whose phonological relation is more tenuous, thanks to the differences in stress and vowel quality. This prediction is borne out experimentally (Pinker, 1999).

Given the status of schemas, activation spreads not only between words, but also from words to the schemas that they are instances of. For instance, activating *widen* triggers not only the word *wide* but also to some degree the -*en* schema (7). These activations reinforce that of *widen*, increasing the processor’s commitment to this as “the word being heard,” and thereby the judgment is faster and/or more robust. (In probabilistic terms such as in O’Donnell, 2015; the independent activity of the parts increases the probability that the word being heard is *widen*.) Such morphological priming is attested in the experimental literature (Baayen and Lieber, 1991; Feldman, 2000; Koester and Schiller, 2008; Smolka et al., 2014).

Again in concurrence with much of the literature, we take it that the course of processing is *opportunistic* or *incremental*, in the sense that information is brought to bear whenever it becomes available (Marslen-Wilson, 1975; Tanenhaus et al., 1995). Thus we expect that in comprehension, phonological information will be available for processing before it activates syntactic and semantic information, and that the aspects of semantics that do not depend on syntax, such as word meaning, may become available before syntax is. Moreover, consistent with a wealth of evidence from the “visual world” paradigm, even visual information can be brought to bear on syntactic parsing, if available in time (Tanenhaus et al., 1995; Huettig et al., 2011). On the other hand, passing activation through interface and relational links does take time, which affects the overall time-course of processing. For example, in comprehension, activation cannot spread to a word’s semantic associates until the word’s own semantics has been activated by its phonology.

On this conception, priming of all sorts amounts to transient enhancement of activation. Identity priming occurs when an activated item does not decay immediately to resting level, so it takes less “energy” to reactivate it. Neighborhood priming occurs by virtue of spreading activation through relational links. Semantic priming is neighborhood priming on the semantic level, which is linked to overall understanding of the current linguistic and nonlinguistic context. Morphological priming, as mentioned above, is enhanced activation of a morphologically complex word through concurrent activation of a schema of which the word is an instance. Finally, since syntactic phrase structure schemas like the transitive verb phrase schema are stored items, we can understand structural (a.k.a. syntactic) priming as identity priming on syntactic treelets, albeit perhaps with different strength and time course from word priming (Bock and Loebell, 1990; Ziegler et al., 2018).

Summing up, the PA/RM approach to linguistic representation has direct counterparts in an account of processing. What is stored in memory is a network of linguistic structures, connected by interface links and relational links of varying strengths, along which activation spreads. Thus the theory of representation (“competence”) and the theory of processing (“performance”) can be brought closely into alignment. Again, it is probably straightforward to incorporate CxG into a similar account of processing. A crucial part would be the addition of relational links, which make possible spreading activation, neighborhood effects, and the ability of schemas to facilitate processing of their instances.

## Beyond Language

Relational morphology focuses on the question of what a speaker stores, and in what form. It can therefore be viewed as a theory of one department of long-term memory. It is intriguing to speculate whether the approach can be extended to other mental faculties, setting as a prospect for future research the possibility of unifying theories of cognition across major components. Our conjecture is that memory is memory is memory – that many of the principles of organization in the language network can be found throughout a variety of cognitive domains. On this view, the differences among domains lie in the formal properties of the mental representations involved and in the interfaces between one domain and the next.

To sum up some of the characteristics we have in mind (for more details and discussion, though equally speculative, see Jackendoff and Audring, 2020, chapter 8):

• Knowledge of language involves a vast lexicon, with tens (or even hundreds) of thousands of items, ranging in size from affixes, through words, to idioms, collocations, and even longer stretches of language such as poems and song lyrics.
• It involves multiple levels of representation – phonological, syntactic, and semantic, coordinated by interface links, with further links to auditory structure (for speech perception), motor representation (for speech production), to orthographical representations, and general conceptual representation.
• Stored items can have hierarchical constituent structure.
• There are both free forms (e.g., *cat*) and bound forms (e.g., *-ous*).
• Stored items can be assembled recursively into larger novel structures, using schemas that allow a generative role.
• Regularities across items are encoded by schemas and relational links among sisters.

For comparison, consider knowledge of music, another universal but culturally varying human activity (Lerdahl and Jackendoff, 1983; Patel, 2008; Schlenker, 2017; Fitch and Popescu, 2019; Mehr et al., 2019). As with language, we can ask what is stored, and in what form, even if what is stored is entirely different from linguistic knowledge. The basic units of musical knowledge are rhythms and pitches (or intervals) rather than phonemes and syntactic categories; there is no semantics in the sense of propositional meaning. One can recognize hundreds if not thousands of popular songs, folk songs, nursery rhymes, hymns, and, for some people, 45-minute symphonies and the like – to the extent that one can identify them immediately on hearing a few random seconds of music, say, upon turning on the radio. Thus one might consider this knowledge a sort of musical lexicon.

One’s knowledge is not just a string of notes: Lerdahl and Jackendoff (1983) and Jackendoff and Lerdahl (2006) show that musical cognition involves multiple hierarchical levels of representation: grouping structure, which is domain-general; metrical structure, which is partially shared with stress systems in language; and tonal hierarchy or “prolongational reduction,” apparently unique to music and cross-culturally widespread. These levels are interconnected by a rich system of (in present terms) interface links; thus the system of music cognition can be considered another sort of PA. In addition, of course, musical structure has to be linked to auditory input. In individuals who sing or play an instrument, musical structure also has to be linked to motor patterns for production. And let us not forget dance as a motor and visual activity closely linked to musical structure.

Stored pieces of music as well as novel pieces partake of these elements of musical structure. However, the distinction between free and bound forms is not so clear in music. Perhaps a candidate for a bound form would be the appoggiatura, a dissonant note, usually on a strong beat, that cannot stand on its own, but has to resolve to a consonance, usually on a weak beat.

Regularities across pieces of music can be captured by schemas (Lerdahl and Jackendoff’s well-formedness and preference rules). Musical schemas, like the basic units and hierarchical representations of music, have little to do with the corresponding components of language. However, just as different languages have different grammars (now a collection of schemas), different genres of music can be characterized in terms of differences in their repertoire of rhythmic, melodic, and harmonic schemas. Music can also establish relational links between individual pieces that share bits of structure (“oh, that song reminds me of this other song!”). More importantly, relational links in music can occur internally to a piece. For instance, the second line of *Happy Birthday* is the same as the first, except that the last two notes are one step higher – and this is part of what makes the song coherent and memorable. Such internal relational links are ubiquitous in music. They occur in language only in special phenomena such as reduplication and rhyme.

The upshot of this admittedly superficial comparison of language and music is that the general organization of memory is shared between the two, while the structures built and stored in memory are of quite different character.

For quite a different domain, consider one’s knowledge of physical objects. There is a vast “lexicon,” containing representations of all the many thousands of objects one knows about: tables, chairs, shoes, shirts, buttons, combs, toothbrushes, pianos, drums, plates, forks, doors, doorknobs, windows, carpets, books, newspapers, cars, trucks, airplanes, roads, rocks, trees, clouds, potatoes, bananas, laptops, televisions, cats, lizards, etc. Each of these involves linked levels of representation: how it looks, how it feels, perhaps how it smells, how you use it (for artifacts – an action representation), and/or how it moves (for animates and vehicles – a different kind of action representation).

Most of these sorts of objects have some hierarchical constituent structure, perhaps along the lines of Marr’s (1982) 3D model or Biederman’s (1987) geons. For instance, a cat has legs, a tail, and a head with eyes, ear, nose, and mouth. A car has wheels and doors; the wheels have tires and hubcaps; the hubcaps may have spokes; the doors have handles; the handles may have keyholes.

There are free and bound items. Most of the objects named above are free. But a stripe is physically bound: there can’t be a stripe without a surface. Holes, cracks, and dents likewise are bound: there can’t be a hole without a volume in which it is situated. A button may be physically free – you can buy individual buttons – but it is functionally bound, in that it only achieves its proper function in the context of a buttonhole and two surfaces to be attached.

There are relational links among items that pick out shared structure. For instance, one can appreciate the similarities in function between radically different forms of bottle openers, lamps, or faucets. And any sort of prototype representation (e.g., a Marr or Biederman model) is in effect a schema. Schemas can pick out generalizations about the layout of parts, for instance windows are normally placed in walls, not floors. Rumelhart (1980) uses the term *schema* in precisely this sense, speaking for instance of a schema for a face, with subschemas for noses and eyebrows and so on.

In short, although this domain of knowledge is built out of qualitatively different components from language, and although it has a different collection of interfaces to other perceptual and cognitive domains, its overall organization, like that of music, can be characterized in terms that are compatible with the PA/RM account of language. Jackendoff and Audring (2020) add two other domains to this list: social cognition and geography/spatial layout. To a considerable extent this collection of domains intersects with Spelke’s (2000) and Carey’s (2009) “domains of core knowledge.”

## Conclusion

On one hand, the PA/RM approach to language can be regarded as a close relative of CxG and especially CxM. Like them, it regards rules of grammar as inhabitants of the extended lexicon (or constructicon); and its schemas are similar in spirit and content to CxG’s constructions. We have stressed two main differences. First, CxG regards constructions as uniformly consisting of systematic pairings of form (phonology and syntax) and function (semantics). PA/RM also incorporates schemas that consist only of a syntactic template, as well as schemas that establish a connection between two or more nonsemantic levels of structure. This richer range of possibilities enables PA/RM to extend into new territories of morphology, syntax, and orthography, as treated in far greater detail in Jackendoff and Audring (2020).

The second major difference between the two frameworks is in the repertoire of relations among lexical items, whether words or schemas. CxG until recently has relied on inheritance as the sole mechanism for relating one item to another: either one is subordinate to the other and inherits structure from it, or they both inherit structure from a more abstract common ancestor. It recently has begun to include tentative paradigmatic links between words and schemas on the same level of representation. PA/RM in contrast places great importance on relational links as a fundamental organizing construct in the lexicon, permitting direct relations between words and between schemas, in both cases pinpointing the regions of correspondence through coindexation. This opens up another broad range of relations among morphological and syntactic patterns that are not available to inheritance.

From a wider perspective, the PA lends itself to a gracefully integrated theory of the language faculty – phonology, morphology, syntax, and semantics – and of the interfaces between language and other cognitive domains. At the same time, the overall character of the network of linguistic knowledge appears to parallel that of other cognitive domains (to the extent that we know anything about them). Speculative though these parallels may be, they are an intriguing step in integrating the language faculty with the rest of the mind. To the degree that the linguistic theory invites such integration, bringing with it a host of deep questions that could not previously be envisioned before, it encourages us to think we are on the right track.

## Data Availability Statement

All datasets presented in this study are included in the article/supplementary material.

## Author Contributions

RJ and JA jointly carried out the research on this project. RJ wrote the article. Both authors contributed to the article and approved the submitted version.

## Conflict of Interest

The authors declare that the research was conducted in the absence of any commercial or financial relationships that could be construed as a potential conflict of interest.
